# Supplementary material for: Predicting visual function by interpreting a neuronal wiring diagram
Source: Nature. 2024 Oct 2;634(8032):113–23. doi: 10.1038/s41586-024-07953-5 (PMC11446822; doi:10.1038/s41586-024-07953-5)
Supplement: Supplementary file 6 — CRF and ERF predictions for individual TmY4 and TmY9 cells. Analogous to Supplementary Data 3, but for TmY target types. Shown are the top four monosynaptic pathways, the strongest pathway passing through each of the top ten intermediary types (ranking from Extended Data Fig. 7), and the trisynaptic pathway Tm1–TmY–Dm3–TmY (see the section entitled Prediction of spatial normalization). [file 41586_2024_7953_MOESM6_ESM.zip › DataS4/TmY9q/720575940634795455.pdf]

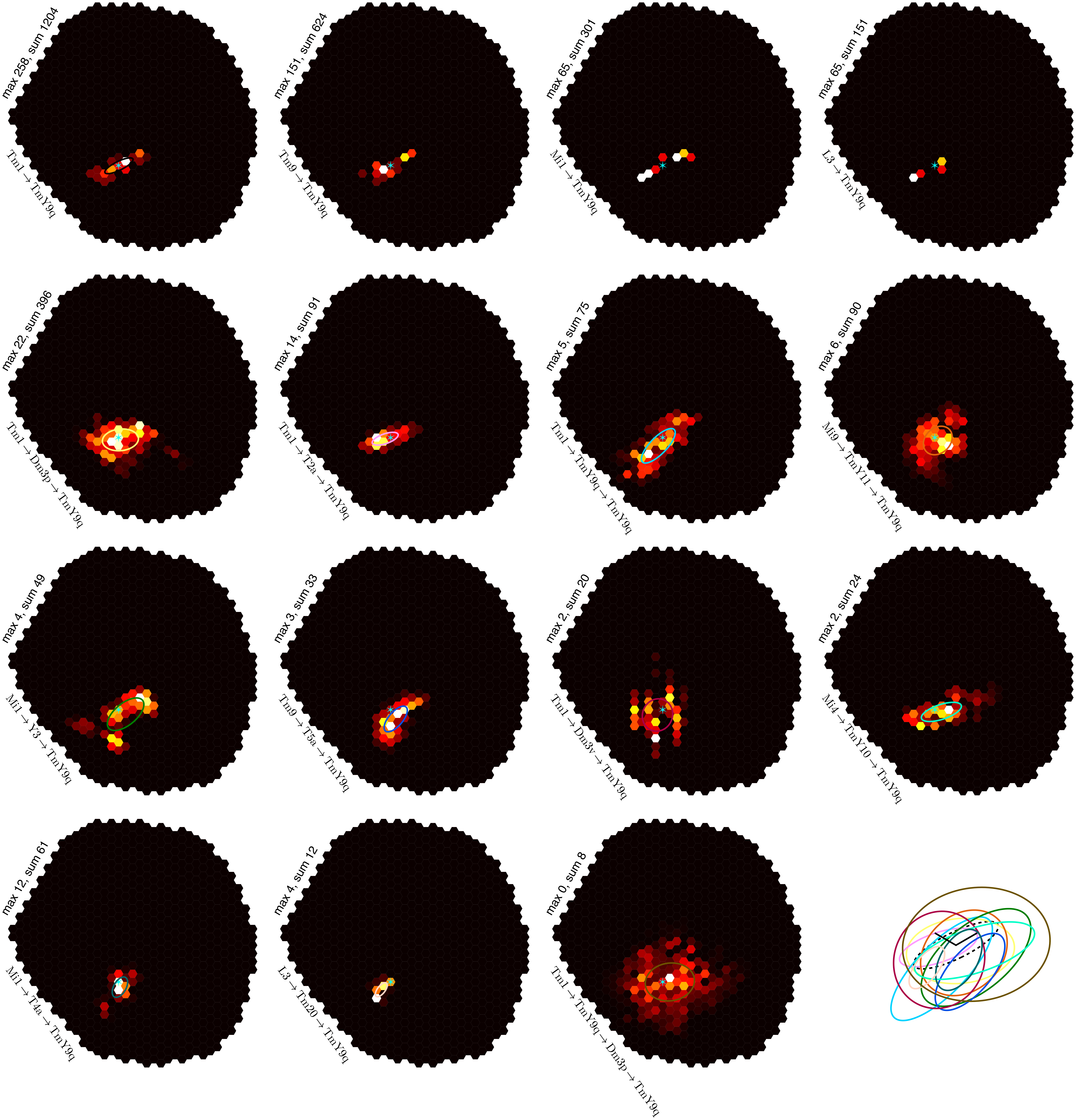
max 258, sum 1204
Tm1 → TmY9q
max 151, sum 624
Tm9 → TmY9q
max 65, sum 301
Mi1 → TmY9q
max 65, sum 151
L3 → TmY9q
max 22, sum 396
Tm1 → Dm3p → TmY9q
max 14, sum 91
Tm1 → T2a → TmY9q
max 5, sum 75
Tm1 → TmY9q → TmY9q
max 6, sum 90
Mi9 → TmY11 → TmY9q
max 4, sum 49
Mi1 → Y3 → TmY9q
max 3, sum 33
Tm9 → T5a → TmY9q
max 2, sum 20
Tm1 → Dm3v → TmY9q
max 2, sum 24
Mi4 → TmY10 → TmY9q
max 12, sum 61
Mi1 → T4a → TmY9q
max 4, sum 12
L3 → Tm20 → TmY9q
max 0, sum 8
Tm1 → TmY9q → Dm3p → TmY9q
